# Antithrombotic treatment and outcome after endovascular treatment and acute carotid artery stenting in stroke patients with atrial fibrillation

**DOI:** 10.1186/s42466-022-00207-7

**Published:** 2022-09-12

**Authors:** Johannes M. Weller, Franziska Dorn, Julius N. Meissner, Sebastian Stösser, Niklas M. Beckonert, Julia Nordsiek, Christine Kindler, Christoph Riegler, Fee Keil, Gabor C. Petzold, Felix J. Bode, the GSR-ET investigators, A. Reich, O. Nikoubashman, J. Röther, B. Eckert, M. Braun, G. F. Hamann, E. Siebert, C. H. Nolte, G. Bohner, R. M. Eckert, J. Borggrefe, P. Schellinger, J. Berrouschot, A. Bormann, C. Kraemer, H. Leischner, M. Petersen, F. Stögbauer, T Boeck-Behrens, S. Wunderlich, A. Ludolph, K. H. Henn, C. Gerloff, J. Fiehler, G. Thomalla, A. Alegiani, J. H. Schäfer, S. Tiedt, L. Kellert, C. Trumm, U. Ernemann, S. Poli, J. Liman, M. Ernst, K. Gröschel, T. Uphaus

**Affiliations:** 1 Division of Vascular Neurology, Department of Neurology, University Hospital Bonn, Venusberg-Campus 1, 53127 Bonn, Germany; 2 Department of Neuroradiology, University Hospital Bonn, Bonn, Germany; 3 Department of Neurology and Center for Stroke Research Berlin, Charité–Universitätsmedizin Berlin, Berlin, Germany; 4 Division of Neuroradiology, University Hospital Frankfurt, Frankfurt, Germany

**Keywords:** Stroke, Endovascular treatment, Mechanical thrombectomy, Carotid artery stenting, Large vessel occlusion, Oral anticoagulation, Antiplatelet therapy, Clinical outcome

## Abstract

**Background:**

Oral anticoagulation (OAC) is the mainstay of secondary prevention in ischemic stroke patients with atrial fibrillation (AF). However, in AF patients with large vessel occlusion stroke treated by endovascular therapy (ET) and acute carotid artery stenting (CAS), the optimal antithrombotic medication remains unclear.

**Methods:**

This is a subgroup analysis of the German Stroke Registry—Endovascular Treatment (GSR-ET), a prospective multicenter cohort of patients with large vessel occlusion stroke undergoing ET. Patients with AF and CAS during ET were included. We analyzed baseline and periprocedural characteristics, antithrombotic strategies and functional outcome at 90 days.

**Results:**

Among 6635 patients in the registry, a total of 82 patients (1.2%, age 77.9 ± 8.0 years, 39% female) with AF and extracranial CAS during ET were included. Antithrombotic medication at admission, during ET, postprocedural and at discharge was highly variable and overall mortality in hospital (21%) and at 90 days (39%) was high.

Among discharged patients (n = 65), most frequent antithrombotic regimes were dual antiplatelet therapy (DAPT, 37%), single APT + OAC (25%) and DAPT + OAC (20%). Comparing DAPT to single or dual APT + OAC, clinical characteristics at discharge were similar (median NIHSS 7.5 [interquartile range, 3–10.5] vs 7 [4–11], *p* = 0.73, mRS 4 [IQR 3–4] vs. 4 [IQR 3–5], *p* = 0.79), but 90-day mortality was higher without OAC (32 vs 4%, *p* = 0.02).

**Conclusions:**

In AF patients who underwent ET and CAS, 90-day mortality was higher in patients not receiving OAC.

*Registration*: https://www.clinicaltrials.gov; Unique identifier: NCT03356392.

**Supplementary Information:**

The online version contains supplementary material available at 10.1186/s42466-022-00207-7.

## Introduction

In acute ischemic stroke patients with cerebral large vessel occlusion and concomitant occlusion or high-grade stenosis of the afferent cervical internal carotid artery, periprocedural acute carotid artery stenting (CAS) is often required during endovascular treatment (ET) [1]. Antithrombotic medication is given to prevent in-stent thrombosis, but no standard antiplatelet regimen has been established. The benefit of dual antiplatelet treatment (DAPT) in CAS was shown in two small trials reporting lower rates of neurological complications [2, 3]. While life-long APT is generally recommended in CAS, the optimal duration of DAPT is unknown, with most guidelines suggesting that DAPT should be administered for at least 4 weeks, as extrapolated from the CREST protocol [1, 4, 5]. The benefit of DAPT vs. single APT has to be counterweighed with an increased risk of intracranial or systemic hemorrhage (major bleeding: 3.4% vs. 1.5%, respectively) [6]. This is particularly relevant in ischemic stroke patients with atrial fibrillation (AF), in whom oral anticoagulation (OAC) is the mainstay of ischemic stroke prevention [7]. Therefore, it has remained unclear which antithrombotic combination is optimal in AF patients treated by ET and CAS.

We performed a secondary analysis of a multi-center stroke registry to describe current clinical practice and outcome of ischemic stroke patients with AF treated with ET and CAS, and to assess the impact of secondary prophylactic treatment including OAC initiated within 4 weeks on clinical outcome in these patients.

## Methods

The GSR-ET (German Stroke Registry–Endovascular Treatment) is a prospective, multicenter registry of patients with acute large vessel occlusion stroke treated by ET. Patients were enrolled between June 2015 and December 2019 from 25 participating stroke centers in Germany. Details have been published previously [8]. The study was centrally approved by the Institutional Review Board of the Ludwig-Maximilians University Munich (protocol no. 689–15) and from local institutional review boards according to local regulations.

All patients with AF and CAS during ET were included in this analysis (n = 82). We describe antithrombotic medication at several timepoints: periprocedural, i.e. during the thrombectomy session; postprocedural, i.e. during the hospital stay after ET; and discharge. Discharge antithrombotic medication was summarized as single APT or DAPT with or without OAC, the latter defined as OAC initiated within 4 weeks.

The primary end point was functional outcome, measured by modified Rankin Scale (mRS) score at 90-day follow-up and analysed as mortality (mRS score 6) and rate of good outcome (mRS scores 0 to 2). Adverse events were assessed during ET as well as pooled from ET until 24 h later and from ET until discharge. Intracranial hemorrhage (ICH) was defined as any hemorrhage on postinterventional imaging during the respective interval.

Descriptive analysis was used for patient characteristics. Group differences were evaluated with Fisher’s exact test, Mann–Whitney *U* test, or unpaired Student *t* test. Multivariable logistic regression was performed to analyze 90-day mortality. In samples < 100 cases, percentages were rounded to full numbers. Significance level was set to α = 0.05. All analyses were performed with R (R version 4.0.3, R core team 2020, package ‘ggsankey’, R Foundation for Statistical Computing, Vienna, Austria. https://www.R-project.org/).

## Results

Among 6635 patients in the registry, we identified 82 (1.2%) acute ischemic stroke patients with AF treated with ET and CAS at 14 centers. The median age was 77.9 years, 39% were female and the median prestroke mRS was 0 (IQR, 0–2). AF was newly diagnosed in 18% and known in 82%, 37% were on OAC at baseline. Further baseline characteristics are shown in Table 1.

**Table 1:** Description of baseline, periprocedural and outcome characteristics

	n = 82
Age, y (SD)	77.9 (8.0)
Female sex, % (n)	39 (32)
Median prestroke mRS (IQR)	0 (0–2)
Median NIHSS (IQR)	16 (11–19)
Median ASPECTS (IQR)	9 (7–10)
*Cardiovascular risk factors, % (n)*	
Hypertension	89 (73)
Diabetes	32 (26)
Dyslipidemia	55 (45)
Atrial fibrillation, known	82 (67)
Atrial fibrillation, newly diagnosed	18 (15)
*Baseline medication, % (n)*	
Antiplatelet therapy	24 (20)
Oral anticoagulation	37 (30)
*Procedural results*	
IVT, % (n)	38 (31)
mTICI ≥ 2b, % (n)	92 (73)
Passages, n (IQR)	1 (1–2)
Median time SO to ADM (IQR)	161 (81–209)^#^
Median time LSW to ADM (IQR)	453.5 (290.5–787.75)^#^
Median time ADM to GRO (IQR)	57 (33–96)
Median time GRO to FLR (IQR)	57 (36–90)
*Periprocedural complications*	
Dissection, perforation, % (n)	5 (4)
ICH, % (n)	2 (2)
Vasospasm, % (n)	1 (1)
Resuscitation, % (n)	2 (2)
Other, % (n)	2 (2)
*Hospital stay*	
Malignant MCA infarction, % (n)	4 (3)
Recurrent stroke, % (n)	13 (11)
In-stent thrombosis, % (n)	4 (3)
ICH, % (n)	15 (12)
Myocardial infarction, % (n)	0
Groin hematoma/aneurysm, % (n)	2 (2)
Other complications, % (n)	28 (23)
Median duration of stay, d (IQR)	8 (5–13)
*Outcome*	
In-hospital mortality, % (n)	21 (17)
Median discharge mRS (IQR)	4 (3–5)
Median discharge NIHSS (IQR)	10 (5–19)
Median mRS at 90 days (IQR)	4 (2–6)
mRS 0–2 at 90 days, % (n)	27 (19)
Mortality at 90 days, % (n)	39 (28)

^#^SO was reported in 62%, LSW in 32%, and no information on time of onset available in 6% (n = 5)

*ASPECTS* Alberta Stroke Program Early CT Score; *IQR* interquartile range; *NIHSS* National Institutes of Health Stroke Scale; *IVT* intravenous thrombolysis; *mTICI* modified Thrombolysis in Cerebral Infarction scale; *mRS* modified Rankin Scale; *SO* symptoms onset; *LSW* last seen well; *ADM* admission; *GRO* groin puncture; *FLR* flow restoration

All cases received extracranial CAS, which was restricted to the internal carotid artery in 95% and extending to the common carotid artery in 5%. CAS was performed due to occlusion in 56% and stenosis in 44%. In 2% of patients, the occlusion resulted from periinterventional dissection, while no case of spontaneous dissection was included. A tandem lesion was present in 62% of patients. CAS was performed before thrombectomy in 40%, afterwards in 41% and in unknown sequence in 18%.

Intravenous thrombolysis was performed in 38%. ET was successful with a modified treatment in cerebral ischemia (mTICI) score of 2b or higher in 92% of patients. Until discharge, recurrent ischemic stroke occurred in 13% and ICH in 15% of patients. Three cases (4%) of in-stent-thrombosis occurred, which required repeat ET in two patients, while the remaining patient died in hospital. Further details are given in Table 1.

**Fig. 1:**
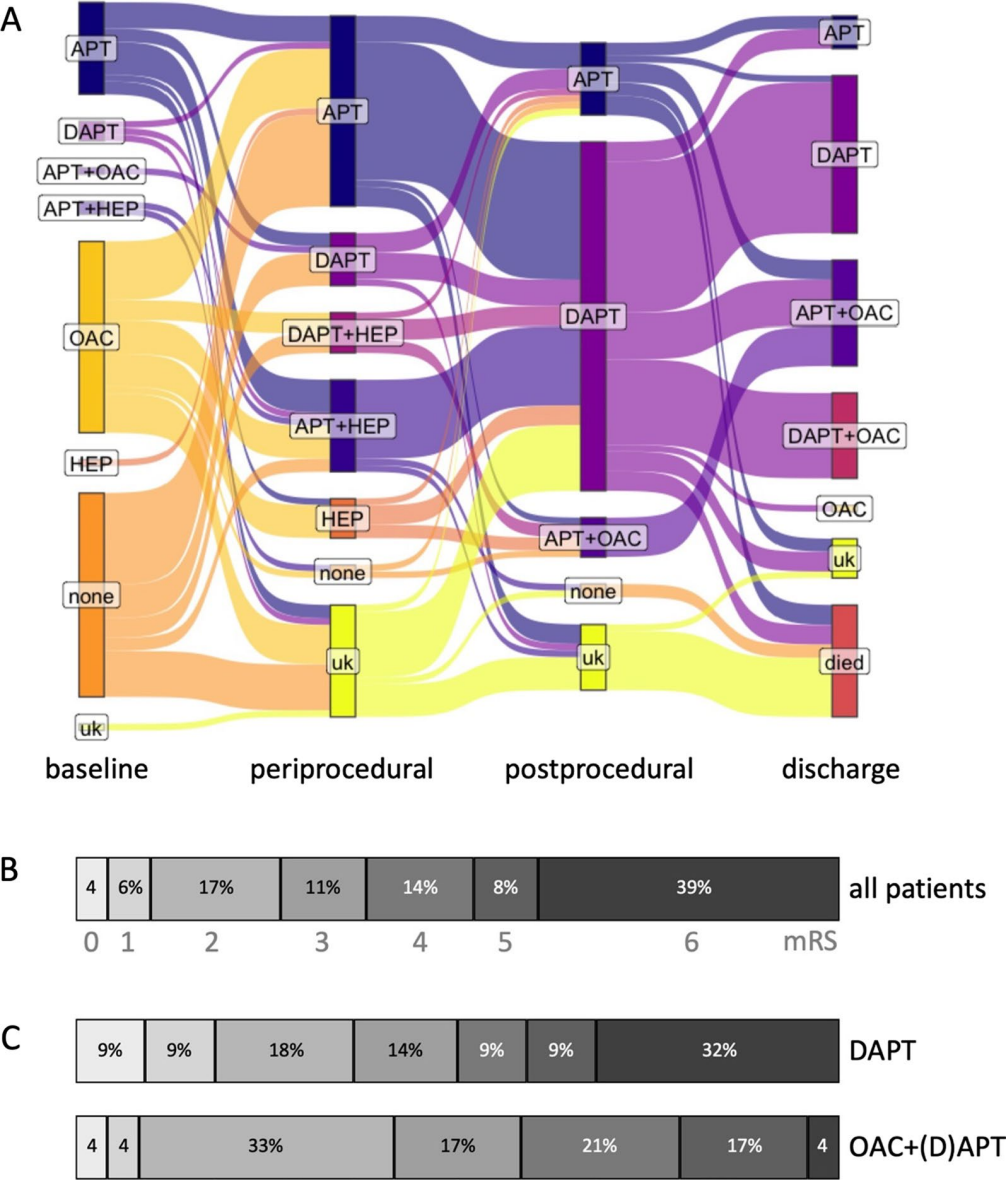
Antithrombotic medication and outcome at 90-day follow-up. **A** Sankey diagram of antithrombotic medication upon baseline, periprocedural, postprocedural and at discharge. **B** Functional outcome of stroke patients with atrial fibrillation after endovascular treatment and carotid artery stenting at 90-day follow-up (n = 82). **C** Comparison of functional outcome for patients discharged with dual antiplatelet therapy (n = 24) compared to patients with oral anticoagulation in addition to antiplatelet therapy (n = 29), see respective groups at ‘discharge’ in Panel A. Abbreviations: *APT, antiplatelet therapy; DAPT, dual antiplatelet therapy; OAC, oral anticoagulation, HEP, therapeutic dosing of heparin; uk, unknown*

Antithrombotic treatment during ET and CAS, postprocedural as well as at discharge was highly variable (Fig. 1, Additional file 1: Table S1). During their hospital stay after the procedure, 65% of patients received DAPT. At discharge, DAPT was the most frequent antithrombotic treatment in 46%, and 34% received OAC in combination with single or dual APT ([D]APT). 12 patients were discharged with (D)APT and recommendation for initiation of OAC at a later time point (Supp. Tabl. 1). Four weeks after discharge, DAPT without OAC decreased to 37%, while 45% of patients received (D)APT in combination with OAC.

At 90-day follow-up, overall mortality was high (39%), but 27% of all patients and 33% of patients with a pre-stroke mRS ≤ 2 reached good functional outcome (Table 1).

### *Comparison of antithrombotic medication in patients discharged after ET and CAS: DAPT vs. OAC* + *(D)APT*

To analyze the impact of secondary prevention with OAC on outcome at 90-day follow-up, we compared patients treated after ET with DAPT only (n = 24) to patients receiving OAC in addition to (single or dual) antiplatelet therapy (OAC + (D)APT, n = 29). The remaining patients were excluded due to missing information on discharge medication (n = 6), insufficient sample size (OAC only: n = 1, APT only: n = 5) and death before discharge (n = 17).

Comparing DAPT to OAC + (D)APT, baseline characteristics were similar with the exception of a trend towards younger age in DAPT patients (76.0 vs. 80.2 years, *p* = 0.053) and higher prevalence of diabetes (38% vs 14%, *p* = 0.06). Intravenous thrombolysis was more frequently performed in patients receiving DAPT for secondary prevention after ET (57 vs. 28%, *p* = 0.048), reflecting the less frequent use of OAC at baseline in that group (13 vs 48%, *p* = 0.008). Intrahospital complications were more frequent in patients where OAC + (D)APT was recommended, but without reaching statistical significance (Table 2). Both groups achieved high rates of recanalization (mTICI ≥ 2b in > 90%) and were similar with regard to NIHSS and mRS both 24 h after stroke and at discharge, as well as with regard to length of hospital stay. Further details on baseline, procedural and outcome characteristics are shown in Table 2.

**Table 2:** Comparison of characteristics and outcome for patients discharged with dual antiplatelet therapy vs. oral anticoagulation in addition to single or dual antiplatelet therapy

	DAPT, n = 24	OAC + (D)APT, n = 29	*p*
Age, y (SD)	76.0 ± 8.2	80.2 ± 7.2	0.053
Female sex, % (n)	33 (8)	24 (7)	0.55
Median prestroke mRS (IQR)	0 (0–1.25)	0 (0–1)	0.87
Median NIHSS (IQR)	14 (9–17)	14 (9–18)	0.96
Median ASPECTS (IQR)	9 (7–10)	10 (8–10)	0.30
*Cardiovascular risk factors, % (n)*			
Hypertension	92 (22)	90 (26)	1.0
Diabetes	38 (9)	14 (4)	0.06
Dyslipidemia	54 (13)	55 (16)	1.0
*Baseline medication, % (n)*			
Antiplatelet therapy	38 (9)	21 (6)	0.23
Oral anticoagulation	13 (3)	48 (14)	0.008
*Procedural results*			
IVT, % (n)	57 (13)	28 (8)	0.048
mTICI ≥ 2b, % (n)	92 (22)	100 (28)	0.21
Passages, n (IQR)	1 (1–2)	1 (1–2)	0.75
Median time SO to ADM (IQR)	166 (111–200.25)^#^	147 (67.5–197.5)^+^	0.70
Median time LSW to ADM (IQR)	300 (241–401.5)^#^	633 (426–888)^+^	0.09
Median time ADM to GRO (IQR)	53.5 (33.25–70.75)	53 (36–107)	0.50
Median time GRO to FLR (IQR)	61.5 (34.5–75.75)	54 (36–91)	0.82
*Hospital stay*			
Malignant MCA infarction, % (n)	0	3 (1)	1.0
Recurrent stroke, % (n)	13 (3)	17 (5)	0.72
In-stent thrombosis, % (n)	8 (2)	0	0.20
ICH, % (n)	4 (1)	17 (5)	0.20
Median NIHSS at 24 h (IQR)	11 (6–15)	10 (8–17.50)	0.82
Median mRS at 24 h (IQR	4 (4–5)	4 (4–5)	0.91
Median discharge NIHSS (IQR)	7.5 (3–10.5)	7 (4–11)	0.73
Median discharge mRS (IQR)	4 (3–4)	4 (3–5)	0.79
Median duration of stay, d (IQR)	9 (7–12.5)	7.5 (6–17.25)	0.75
*Outcome*			
Median mRS at 90 days (IQR)	3.5 (2–6)	3 (2–4)	0.38
mRS 0–2 at 90 days, % (n)	36 (8)	42 (10)	0.77
Mortality at 90 days, % (n)	32 (7)	4 (1)	0.020

^#^SO was reported in 50%, LSW in 42%, no information on time of onset available in 8% (n = 2)

^+^SO was reported in 66%, LSW in 28%, no information on time of onset available in 7% (n = 2)

*DAPT* dual antiplatelet therapy; *OAC* oral anticoagulation; *(D)APT* single or dual antiplatelet therapy; ASPECTS Alberta Stroke Program Early CT Score; *IQR* interquartile range; *NIHSS* National Institutes of Health Stroke Scale; *IVT*, intravenous thrombolysis; *mTICI* modified Thrombolysis in Cerebral Infarction scale; *mRS* modified Rankin Scale; *SO* symptoms onset; *LSW* last seen well, *ADM* admission; *GRO* groin puncture; *FLR* flow restoration

At 90-day follow-up, the rate of good functional outcome was similar for DAPT compared to OAC + (D)APT (36 vs 42%, *p* = 0.77), but late mortality was significantly higher in the DAPT group (32 vs. 4%, *p* = 0.020). This was confirmed by logistic regression adjusted for age and discharge mRS (*p* = 0.021).

## Discussion

In this study, we have analyzed patients with large vessel occlusion stroke and AF receiving treatment with ET and CAS. Importantly, good functional outcome after 3 months (mRS ≤ 2) was achieved in 27% and overall mortality was high with almost 39%, thus outcome was worse compared to the whole GSR-ET cohort, where good outcome was reported in 37% of patients and mortality was 29%. [9]

Optimal antithrombotic treatment in patients with acute CAS and AF is unknown and current guidelines provide little recommendations. DAPT is the mainstay of antithrombotic treatment after CAS and usually recommended for at least 30 days. [1, 4, 5] However, OAC is necessary in patients with AF as DAPT is clearly inferior for stroke prevention [10]. We are not aware of any trials addressing optimal treatment in this setting of stenting and AF. There are some retrospective data on antithrombotic treatment in elective CAS and AF: A small study of 31 patients reported fewer adverse events (0 vs. 2 events) in direct oral anticoagulation (DOAC) + APT compared to vitamin K-antagonists (VKA) + DAPT [11], and another cohort of 32 patients receiving a short course of periprocedural triple therapy and DOAC + APT upon discharge reported no thrombosis or bleeding within 30 days. [12] In an insurance database analysis, APT was associated with higher mortality (26.7% in 2 years) compared to DOAC + APT or DOAC (21.2% and 22.9%, respectively) [13]. There were numerically fewer ischemic strokes and more frequent bleeding events in DOAC + APT, but the first month after CAS was not included. A recent retrospective cohort of 91 patients reported an increased bleeding rate of 23.8% in the first month with triple therapy (DAPT+OAC, 23.8%) compared to DAPT (4%) or DOAC + APT (0%), while there was no thromboembolic event in the triple therapy group compared to 1 event each in DAPT or DOAC + APT [14]. Reflecting the difficulty of drug choice, a wide variety of antithrombotic regimes was given in our cohort, ranging from APT to dual (APT + OAC) or triple therapy. Importantly, 90-day mortality was significantly higher if patients were treated only with DAPT after discharge as compared to OAC in addition to single or dual APT.

As far as this is comparable, in patients with lower extremity stenting and AF, triple therapy is discouraged with the exception of high risk lesions or stenting below the knee, depending on bleeding and ischemia risk [15]. In acute coronary syndrome and AF, an initial peri-/postprocedural period of triple therapy is recommended and may be extended for up to 1 month, but dual treatment may be considered in excessive bleeding risk [16, 17]. In a recent meta-analysis, APT + OAC reduced the risk of bleeding when compared to triple treatment, but the risk of death or ischemia remains unclear [18]. While the severity of stent occlusion might be similar in myocardial infarction and ischemic stroke, the local bleeding risk after cerebral infarction is higher. Only one patient in our cohort was treated with DAPT + therapeutic heparin during the initial postprocedural period, as early triple therapy is mostly assumed to cause excessive bleeding risk in cerebral infarction. Of note, the optimal timing of OAC (re-)initiation is unknown in ischemic stroke patients with AF even in absence of CAS, and mainly based on expert opinions. [7, 19, 20]

Regarding the choice of OAC treatment, all patients were treated with DOACs and none with VKA, which is in line with current recommendations [7]. The use of DOAC over VKA in the setting of dual or triple therapy is also supported by studies in AF patients with acute coronary syndrome, where DOAC + APT was superior or non-inferior to VKA + DAPT. [21, 22]

In some cases, medication deviated from current recommendations, e.g. discharge with single APT [1, 5], and one patient was discharged with OAC only, but there were too few cases for further analysis and confounding by indication seems plausible.

Our study has all limitations of a retrospective analysis of a prospectively collected registry. Medication regimes were evaluated based on therapy during the hospital stay and recommendations upon discharge, but changes might have been introduced later. Comparing patients with DAPT to OAC + (D)APT, no differences were detected with regard to periprocedural characteristics, intrahospital complications, length of hospital stay and discharge condition. Nevertheless, confounding by indication is an important limitation. Furthermore, information on the cause of death was not available.

We included acute ischemic stroke patients with AF, who were treated with ET and CAS. While retrospective data suggest a benefit of CAS in patients without AF treated with ET for tandem occlusions [23, 24], evading stenting might be feasible especially in patients with AF. In this context, the results of randomized controlled trials (TITAN, EASI-TOC) will hopefully help determine the best approach. [25, 26]

## Conclusion

In summary, the optimal antithrombotic treatment in AF patients with acute ischemic stroke, ET and CAS is unknown. While APT or DAPT is necessary to maintain stent patency, our data indicate that (re-)initiation of OAC should not be omitted. Randomized controlled studies are warranted to define the optimal antithrombotic regime in this setting.

## Supplementary Information

**Additional file 1.** Antithrombotic medication and outcome at 90-day follow-up.

## Data Availability

The datasets used and/or analyzed in the current study are available from the corresponding author on reasonable request.

## Abbreviations

DAPT: Dual antiplatelet therapy
OAC: Oral anticoagulation
APT: Antiplatelet therapy
ASPECTS: Alberta Stroke Program Early CT Score
IQR: Interquartile range
NIHSS: National Institutes of Health Stroke Scale
IVT: Intravenous thrombolysis
mTICI: Modified Thrombolysis in Cerebral Infarction scale
mRS: Modified Rankin Scale
SO: Symptoms onset
LSW: Last seen well, ADM: admission
GRO: Groin puncture
FLR: Flow restoration
AF: Atrial fibrillation
ET: Endovascular therapy
CAS: Carotid stenting
