# Supplementary material for: Antithrombotic treatment and outcome after endovascular treatment and acute carotid artery stenting in stroke patients with atrial fibrillation
Source: Neurol Res Pract. 2022 Sep 12;4:42. doi: 10.1186/s42466-022-00207-7 (PMC9465921; doi:10.1186/s42466-022-00207-7)
Supplement: Supplementary file 1 — Additional file 1. Antithrombotic medication and outcome at 90-day follow-up. [file 42466_2022_207_MOESM1_ESM.docx]

**Additional file 1: Table S1 Complete list of employed antithrombotic medications.**

Abbreviations: *DOAC, direct oral anticoagulant; ASS, aspirin; VKA, vitamin K antagonist.*

| **baseline (n)** | **periprocedural (n)** | **postprocedural (n)** | **discharge (n)** |
| --- | --- | --- | --- |
| paused (7) | ASS (18) | ASS (7) | ASS (4) |
| ASS (13) | clopidogrel (3) | clopidogrel (4) | clopidogrel (2) |
| ASS, clopidogrel (2) | eptifibatid (1) | ASS, clopidogrel (50) | - switch to clopidogrel, DOAC after 2 weeks (1) |
| ASS, ticagrelor (1) | tirofiban (7) | ASS, ticagrelor (3) | ASS, clopidogrel (28) |
| ASS, VKA (1) | heparin (6) | ASS, DOAC (2) | - switch to ASS, DOAC after 3 weeks (1) |
| ASS, heparin (1) | ASS, heparin (12) | clopidogrel, DOAC (4) | - switch to ASS, DOAC after 4 weeks (1) |
| clopidogrel (1) | ASS, clopidogrel (1) | none (2) | - switch to ASS, DOAC after 6 weeks (4) |
| clopidogrel, heparin (1) | ASS, ticagrelor (3) | unknown (10) | - switch to ASS, DOAC after 12 weeks (1) |
| DOAC (19) | ASS, tirofiban (3) |  | - switch to clopidogrel, DOAC after 1 week (1) |
| VKA (10) | clopidogrel, heparin (1) |  | - switch to clopidogrel, DOAC after 2 weeks (1) |
| heparin (1) | tirofiban, heparin (1) |  | - switch to clopidogrel, DOAC after 4 weeks (1) |
| none (24) | ASS, clopidogrel, eptifibatid (1) |  | ASS, prasugrel (1) |
| unknown (1) | ASS, clopidogrel, heparin (4) |  | ASS, ticagrelor (1) |
|  | ASS, tirofiban, heparin (2) |  | - switch to ASS, DOAC after 2 weeks (1) |
|  | none (2) |  | ASS, DOAC (4) |
|  | unknown (17) |  | - switch to DOAC after 6 weeks (1) |
|  |  |  | clopidogrel, DOAC (6) |
|  |  |  | ASS, clopidogrel, DOAC (12) |
|  |  |  | DOAC (1) |
|  |  |  | unknown (6) |
|  |  |  | *dead (17)* |
